# Protein Kinase C Inhibition Mediates Neuroblast Enrichment in Mechanical Brain Injuries

**DOI:** 10.3389/fncel.2018.00462

**Published:** 2018-11-27

**Authors:** Francisco García-Bernal, Noelia Geribaldi-Doldán, Samuel Domínguez-García, Manuel Carrasco, Maribel Murillo-Carretero, Antonio Delgado-Ariza, Mónica Díez-Salguero, Cristina Verástegui, Carmen Castro

**Affiliations:** ^1^Área de Fisiología, Facultad de Medicina, Universidad de Cádiz, Cádiz, Spain; ^2^Instituto de Investigación e Innovación en Biomedicina de Cádiz (INIBICA), Universidad de Cadiz, Cádiz, Spain; ^3^Departamento de Anatomía, Facultad de Medicina, Universidad de Cádiz, Cádiz, Spain

**Keywords:** PKC, neurogenesis, brain injuries, neuroblasts, neuronal differentiation

## Abstract

Brain injuries of different etiologies lead to irreversible neuronal loss and persisting neuronal deficits. New therapeutic strategies are emerging to compensate neuronal damage upon brain injury. Some of these strategies focus on enhancing endogenous generation of neurons from neural stem cells (NSCs) to substitute the dying neurons. However, the capacity of the injured brain to produce new neurons is limited, especially in cases of extensive injury. This reduced neurogenesis is a consequence of the effect of signaling molecules released in response to inflammation, which act on intracellular pathways, favoring gliogenesis and preventing recruitment of neuroblasts from neurogenic regions. Protein kinase C (PKC) is a family of intracellular kinases involved in several of these gliogenic signaling pathways. The aim of this study was to analyze the role of PKC isozymes in the generation of neurons from neural progenitor cells (NPCs) *in vitro* and *in vivo* in brain injuries. PKC inhibition *in vitro*, in cultures of NPC isolated from the subventricular zone (SVZ) of postnatal mice, leads differentiation towards a neuronal fate. This effect is not mediated by classical or atypical PKC. On the contrary, this effect is mediated by novel PKCε, which is abundantly expressed in NPC cultures under differentiation conditions. PKCε inhibition by siRNA promotes neuronal differentiation and reduces glial cell differentiation. On the contrary, inhibition of PKCθ exerts a small anti-gliogenic effect and reverts the effect of PKCε inhibition on neuronal differentiation when both siRNAs are used in combination. Interestingly, in cortical brain injuries we have found expression of almost all PKC isozymes found *in vitro*. Inhibition of PKC activity in this type of injuries leads to neuronal production. In conclusion, these findings show an effect of PKCε in the generation of neurons from NPC *in vitro*, and they highlight the role of PKC isozymes as targets to produce neurons in brain lesions.

## Introduction

Acute or chronic injuries cause damage in the adult brain producing neuronal death and irreversible cognitive deficits or sensory-motor alterations (Blennow et al., 2012). Yet no effective treatment is currently available to compensate neuronal loss, new therapeutic strategies are being developed most of them aimed to enhance endogenous repair mechanisms that require the generation of neurons from neural stem cells (NSCs).

Physiologically, adult neurogenesis mainly occurs in specific brain areas such as the dentate gyrus (DG) of the hippocampus and the subventricular zone (SVZ; Gage et al., 1995; Doetsch et al., 1997). NSC in these areas exhibit a low division rate and they generate highly proliferative intermediate progenitors: neural progenitor cells (NPCs). These cells have the capacity to generate neuronal or glial progenitors (neuroblasts), which after a differentiation process produce mature neurons (Alvarez-Buylla and Garcia-Verdugo, 2002; Goldman, 2003) or mature glial cells (astrocytes or oligodendrocytes), respectively.

It is now widely accepted that CNS injuries of different origins: mechanical, vascular (ischemic or hemorrhagic) and neurodegenerative promote neurogenesis in both the DG and the SVZ. The proliferation of NPC within the DG and SVZ generates neuroblasts, which ultimately attempt to migrate towards the site of injury (Liu et al., 1998; Fallon et al., 2000; Jin et al., 2001). Nevertheless, SVZ and DG are not the only source of the NPC found in lesions. In this sense, evidences show that local cortex-residing NSC produce NPC that proliferate and differentiate in response to cortical injury in rodents (Magavi and Macklis, 2002; Buffo et al., 2008). According to these facts, it would be reasonable to expect a significant neuronal replacement rate in response to brain injuries. Notwithstanding this rate has been shown to be very limited and variable depending on factors such as injury size or location of the affected area (Parent et al., 1997; Liu et al., 1998; Jin et al., 2001; Arvidsson et al., 2002; Nakatomi et al., 2002; Teramoto et al., 2003; Romero-Grimaldi et al., 2011; Saha et al., 2013; Moraga et al., 2014). The limited capacity of injured brain tissue to generate new neurons is governed by the activation of glial cells within the injury creating a glial scar formed by glial cells generated from local or migrating NPC (Seidenfaden et al., 2006), local proliferative and activated astrocytes (Liu et al., 2000; Buffo et al., 2008), microgial cells (Rhodes et al., 2003), oligodendrocyte progenitors and fibroblasts (Sofroniew and Vinters, 2010; Kawano et al., 2012). Glial scar forming cells secrete a complex extracellular matrix, which controls signaling events, reduce neuronal survival (Marín-Teva et al., 2011; Benarroch, 2013) and impairs migration towards the injury (Fawcett and Asher, 1999; Silver and Miller, 2004; Carulli et al., 2005). As a result, a gliogenic/non-neurogenic environment is created surrounding the injury, which prevents the generation of neuros from NSC activated within the perilesional area, neuroblast migration from neurogenic regions, and neuroblast survival (Fawcett and Asher, 1999; Silver and Miller, 2004; Carulli et al., 2005; Marín-Teva et al., 2011; Benarroch, 2013).

Recent reports aimed to understand the cellular and molecular mechanisms involved in the generation of this gliogenic/non-neurogenic background highlight the key role of neurogenic signaling molecules such as noggin (Lim et al., 2000) or gliogenic signaling pathways such as those initiated by the epidermal growth factor (EGF) receptor (EGFR; Kuhn et al., 1997; Gonzalez-Perez et al., 2009). Evidences point out at the mission of kinases of the protein kinase C (PKC) family on stimulating the production of signaling molecules such as TGFα and neuregulin, (Dang et al., 2011, 2013), which lead to gliogenesis or neurogenesis respectively (Ghashghaei et al., 2006; Romero-Grimaldi et al., 2011). Ten isozymes comprise the PKC family, which share a highly conserved catalytic domain linked to a more divergent amino-terminal regulatory domain. Based on their specific activators, PKCs are subdivided into three subfamilies: the classical or conventional PKCs (cPKCs α, β and γ), activated by Ca^2+^, diacylglycerol (DAG) and phosphatidylserine (PS); the novel PKCs (nPKCs δ, ε, θ and η), activated by DAG and PS; and the atypical PKCs (aPKCsζ and λ), which require phosphatydilserine and protein-protein interactions (Rosse et al., 2010).

Several members of the PKC family are expressed in neurogenic regions (Minami et al., 2000) and participate in distinct signaling cascades initiated by growth factors (GFs), often determining GF specificity (Corbit et al., 2000). General activation of PKC promotes proliferation of NPC *in vitro* and *in vivo* in the SVZ and DG of mice (Geribaldi-Doldán et al., 2015). In addition, atypical PKC have been involved in the NSC-to-neuron transition during development, and in the adult brain (Wang et al., 2012), on the contrary, novel PKCε, activation is crucial for the astrocytic differentiation of NPCs (Steinhart et al., 2007). Thus, it is reasonable to hypothesize that additional isozymes of the PKC family could be implicated in different steps implicated in adult neurogenesis, like NSC self-renewal, proliferation, survival or neuronal differentiation.

We report in here that general inhibition of PKC isozymes promotes differentiation of NPCs towards a neuronal lineage *in vitro* in NPC cultures. We show that several PKC isozymes are expressed in NPC cultures under differentiation conditions. However, not all of them participate in neuronal differentiation. Particularly, we show that *in vitro* inhibition of classical PKC has no effect on NPC differentiation whereas inhibition of the novel PKCε promotes neuronal differentiation *in vitro*. On the contrary, inhibition of PKCθ slightly reduces glial cell differentiation, but most importantly the inhibition of PKCθ reverts the neurogenic effect of PKCε inhibition. Moreover, we have elucidated that several PKC isozymes in mechanical cortical brain lesions and that infusion of a general PKC inhibitor locally within the lesion facilitates the generation of neurons around the perilesional area.

## Materials and Methods

### Reagents

Gö6850 or Bisindolylmaleimide I, the broad-spectrum PKC inhibitor, was purchased from Calbiochem (San Diego, CA, USA), dissolved in dimethyl sulfoxide (DMSO) and diluted to a final concentration of 0.5 μM with sterile phosphate-buffered saline (PBS) prior to animal administration or in culture medium for* in vitro* analysis. Gö6976, the inhibitor for classical PKC, was purchased from Calbiochem (San Diego, CA, USA), dissolved in DMSO and diluted to a final concentration of 0.16 μM in culture medium for *in vitro* experiments. Other products, unless otherwise indicated, were purchased from Sigma-Aldrich (St. Louis, MO, USA).

### Animal Subjects

Two-month-old adult male CD1 mice were used for *in vivo* experiments. Seven-day postnatal (P7) CD1 mice were used for the isolation of NPC from the SVZ. Animals were housed under controlled conditions of temperature (21–23°C) and light (LD 12:12) with free access to food (AO4 standard maintenance diet; SAFE, Épinay-sur-Orge, France), and water. The study was approved by the Ethics Committee at Consejería de Agricultura, Pesca y Medio Ambiente, Junta de Andalucía, Spain following Guidelines of the European Union Council (2010/63/EU), and following the Spanish regulations (65/2012 and RD53/2013) for the use of laboratory animals.

### SVZ Cell Isolation and Culture

NPC were obtained from the SVZ of P7 mice following the same procedure described in Rabaneda et al. (2008), and were cultured in defined medium (DM), composed of Dulbecco’s modified Eagle’s medium/F-12 nutrient mixture (DMEM/F-12) plus 1 mg/L gentamicin, 200 mM glutamine and the B27 supplement (Invitrogen; Carlsbad, CA, USA). EGF (20 ng/ml) and basic fibroblast growth factor (bFGF, 10 ng/ml), both from PeproTech (Frankfurt, Germany), were added to DM for NPC culture expansion in the form of neurospheres, but were withdrawn from the media for NPC differentiation experiments. Culture media and reagents, unless otherwise indicated, were from GIBCO^1^.

### NPC Cultures Differentiation, and Immunocytochemistry

NPCs were cultured as neurospheres and at the time of the differentiation experiments cells were disaggregated from the neurospheres and adhered onto poly-L-ornithine-coated 1.8-mm-diameter round coverslips in DM media without GFs. Cells were allowed to differentiate for 72 h and were then fixed with 4% paraformaldehyde (PFA) and processed for GFAP and β-III-tubulin immunodetection as previously described (Rabaneda et al., 2008). Antibodies used were: mouse monoclonal anti-β-III-tubulin (1:1,000; Cell Signaling Technology, Boston, MA, USA), rabbit polyclonal anti-GFAP (1:3,000; Dako, Hamburg, Germany), rabbit polyclonal anti-NG2 (1:400; Merk Millipore, Billerica, MA, USA); rabbit polyclonal anti-s100β (1:500; Abcam, Cambridge, UK); mouse monoclonal anti-nestin (1:200; Merk Millipore, Billerica, MA, USA). The secondary antibodies were: goat anti-mouse IgG labeled with AlexaFluor 594 and donkey anti-rabbit IgG labeled with AlexaFluor 488 (1:1,000; Invitrogen, Carlsbad, CA, USA). Total nuclei were counterstained for 10 min with 0.1 mg/L DAPI. Cells positive for β-III-tubulin or GFAP were counted under a BX60 epifluorescence microscope (Olympus, Hamburg, Germany) or under a confocal microscope Olympus Flourview FV 1000 and expressed as percentage of total number of cells. Quantification was performed in 12 predetermined visual fields per coverslip. Experiments were repeated three times with triplicate samples, and results were expressed as the Mean ± SEM.

### NPC Cultures Transfection

Neurosphere cells were disaggregated and adhered onto poly-L-ornithine-coated 1.8-mm-diameter round coverslips in DM media without GF and without antibiotics. Four hours later, cells were transfected with SMARTpool siRNA or a control siRNA pool (Thermo Scientific Dharmacon, Lafayette, CO, USA^2^). Lipofectamine 2000 (Invitrogen, Carlsbad, CA, USA) was used for transfection of these siRNA pools according to manufacturer’s instructions. Cells were allowed to differentiate for 72 h, with a medium change after the first 24 h to eliminate lipofectamine.

### Mechanical Lesions in Brain Cortex

Unilateral lesions were performed in the right brain cortex of adult mice anesthetized with an intraperitoneal injection of a 100 mg/kg ketamine and 20 mg/Kg xylazine cocktail. Animals were placed in a stereotaxic frame (Kopf Instruments), a small craniotomy was performed at +1.4 mm rostral and +1.5 mm lateral to Bregma allowing a controlled mechanical lesion in the underlying primary motor cortex penetrated 1 mm below the bone surface with a manually driven drill (0.7 mm diameter).

### RNA Isolation, Reverse Transcription and Real-Time Quantitative PCR

Total RNA isolation from differentiated cells, the injured and the intact cortex, reverse transcription and RT-qPCR to relatively quantify the expression of the different isozymes of PKC were performed as previously described (Romero-Grimaldi et al., 2011). The housekeeping transcript used was 18S rRNA. All PCR reaction within each experiment was run in duplicates. Amplification specificity was confirmed by melting-curve analysis of the PCR products. The relative expression of each mRNA was calculated as 2^−ΔCt^, where ΔCt = Ct (target mRNA) − Ct (18S). No signal was detected in non-template or non-RT controls.

Primer sequences (5′–3′) and annealing temperatures were the following: for PKCα, FW: TGAATCCTCAGTGGAATGAGT, RW: GGTTGCTTTCTGTCTTCTGAA, 55°C; for PKCβ, FW: CCCGAAGGAAGCGAGGGCAATGAAG, RW: AGTTCATCTGTACCCTTCCGCTCTG, 63°C; for PKCγ, FW: TGAGAGAGTGCGGATGGGCCCC, RW: GCAGGCGTCCTGGGCTGGCACC, 65°C; for PKCδ, FW: GAGGCCTTGAACCAAGTGACCC, RW: CTTGCCATAGGTCCAGTTGTTG, 57°C; for PKCε, FW: CCCATCTGAAGACGACCGATCC, RW: CGGTTGTCAAATGACAAGGCC, 60°C; for PKCθ, FW: CCATGTCACCGTTTCTTCGAATC, RW: TCTGCCCATTTTCTGATTCC, 56°C; for PKCη, FW: ATGGCCACGTACCTGAGGCAGC, RW: GGACGACGCAGGTGCACACTTGG, 65°C; for PKCλ, FW: CGTTGGGAGCTCTGACAATC, RW: ACCTGCTTTTGCTCCATCATG, 55°C; for PKCζ, FW: AGGAGAAGAGTACGGGTTCAGC, RW: GTGTTCATGTCAGGGTTGTCCG, 57°C.

### Studies Describing Neurogenic Response in Mechanical Lesions

In order to study neurogenesis and gliogenesis in mechanical lesions as well as in the SVZ and DG of injured mouse brains, mice were injured using the above-mentioned procedure and left for 14 days before sacrifice. Mice were sacrificed by a lethal dose of barbiturate and perfused by ascending aorta. Subsequently brains were processed for post-mortem studies as described below.

### Studies Describing Migration in Response to Mechanical Lesions

In a different set of experiments, aimed to study migration of progenitors from neurogenic regions towards the injured area, mice received bromodeoxyuridine (BrdU) injections on days 6, 5 and 4 before performing the injury. On the day of injury, animals were implanted osmotic minipumps to allow the continuous delivery of Gö6850 or vehicle, until they were sacrificed 14 days post injury (dpi; see chronogram in Figure 9B). This experimental design allowed the clearance of most of the BrdU from the mouse body before the cortex was injured. This strategy granted BrdU labeling of NPC in the SVZ but not in the injured tissue (since injury was not performed when BrdU was administered). Mice with different treatments (vehicle; *n* = 6 and Gö6850; *n* = 6) were sacrificed on day 14 after injury.

**Figure 1:**
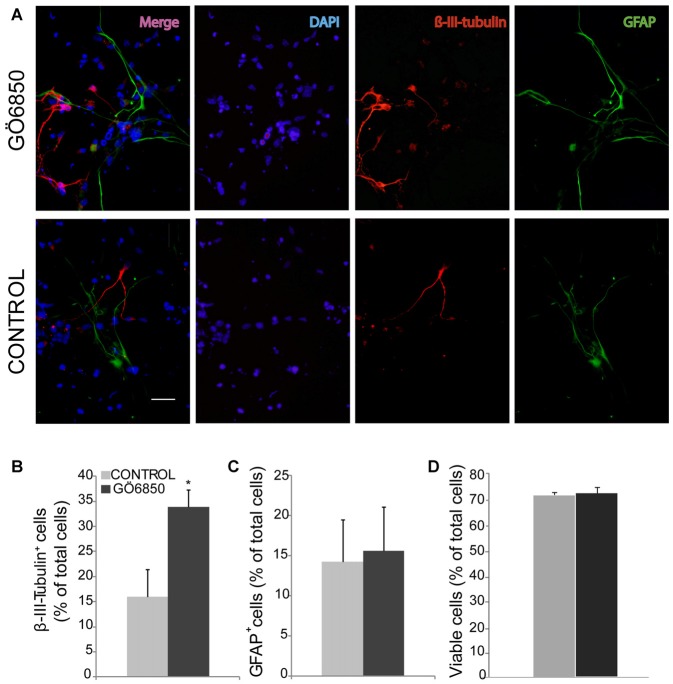
Inhibition of protein kinase c (PKC) activity promotes differentiation of neural progenitor cells (NPCs) to neuroblasts. **(A)** Representative fluorescence microphotographs of subventricular zone (SVZ)-derived cultured neural precursors that had been treated with either diluent (control) or the general PKC inhibitor Gö6850. Cells were grown in the absence of growth factor and allowed to differentiate for 72 h after treatment and then fixed. Neuronal cells were identified by the immunocytochemical detection of β-III-tubulin (red); glial cells were identified by the immunocytochemical detection of GFAP (green) and total nuclei were counterstained with DAPI (blue). Scale bar = 50 μm. **(B)** Graph represents the percentage of total cells (detected by DAPI nuclear staining) that were positive for β-III-tubulin expression. **(C)** Graph represents the percentage of total cells (detected by DAPI nuclear staining) that were positive for GFAP expression. **(D)** Graph represents the percentage of viable cells after treatment as a percentage of total cells (detected by DAPI nuclear staining). Results show a statistically significant increase in the percentage of β-III-tubulin^+^ cells whereas no change of GFAP^+^ cells is observed in the presence of the inhibitor. Data are the Means ± SEM; *n* = 3 independent experiments performed in triplicates. Statistical analysis: *p < 0.05 by Student’s *t*-test comparing with the control group.

**Figure 2:**
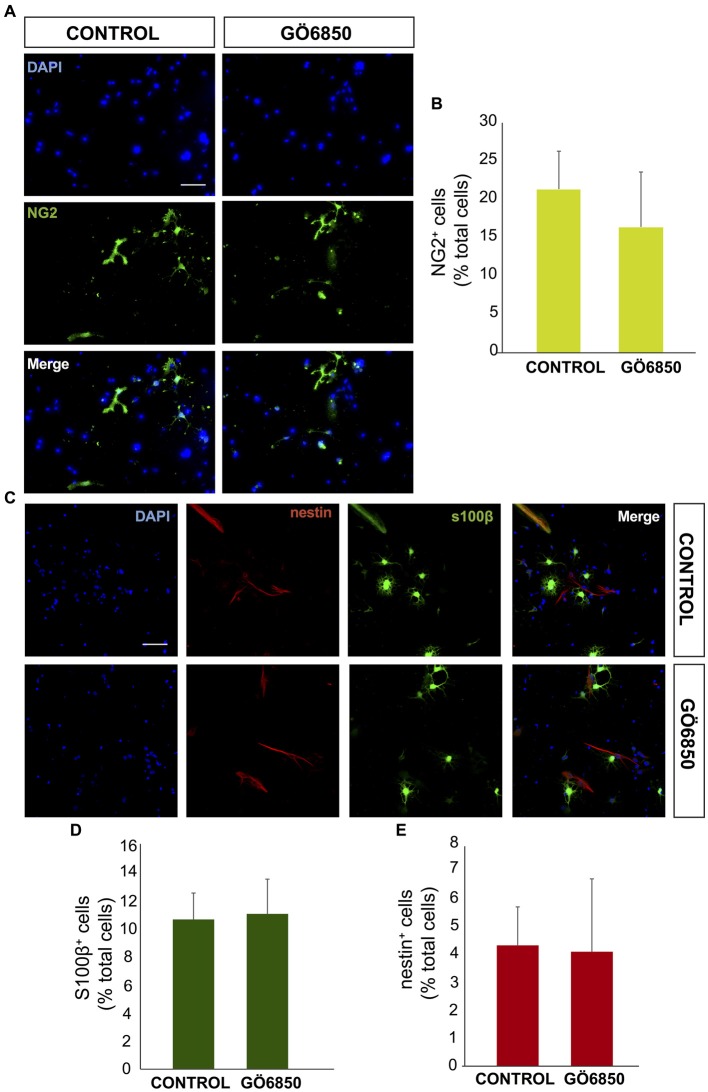
General inhibition of PKC activity has no effect on oligodendrocytes differentiation. **(A)** Representative fluorescence microphotographs of SVZ-derived cultured neural precursors that had been treated with either diluent (control) or the general inhibitor of PKC Gö6850. Cells were grown in the absence of growth factor and allowed to differentiate for 72 h and then fixed. Oligodendrocytes precursors were identified by the immunocytochemical detection of NG2 (green) and total nuclei were counterstained with DAPI (blue). Scale bar = 50 μm. **(B)** Graph represents the percentage of total cells (detected by DAPI nuclear staining) that were positive for NG2 expression. Results show no statistically significant changes in the percentage of NG2^+^ cells induced by the treatment. **(C)** Representative confocal microphotographs of SVZ-derived cultured neural precursors that had been treated with either diluent (control) or the general inhibitor of PKC Gö6850. Astrocytes were identified by the immunocytochemical detection of s100-β (green) and total nuclei were counterstained with DAPI (blue), neural progenitor precursors were identified by the immunocytochemical detection of nestin (red). Scale bar = 50 μm. **(D)** Graph represents the percentage of total cells (detected by DAPI nuclear staining) that were positive for the astrocyte marker s100-β. **(E)** Graph represents the percentage of total cells (detected by DAPI nuclear staining) that were positive for the neural precursor marker nestin. Results show no statistically significant changes in the percentage of s100-β or nestin cells induced by the treatment. Data are the Means ± SEM; *n* = 3 independent experiments performed in triplicates. Statistical analysis: *p < 0.05 by Student’s *t*-test comparing with the control group.

**Figure 3:**
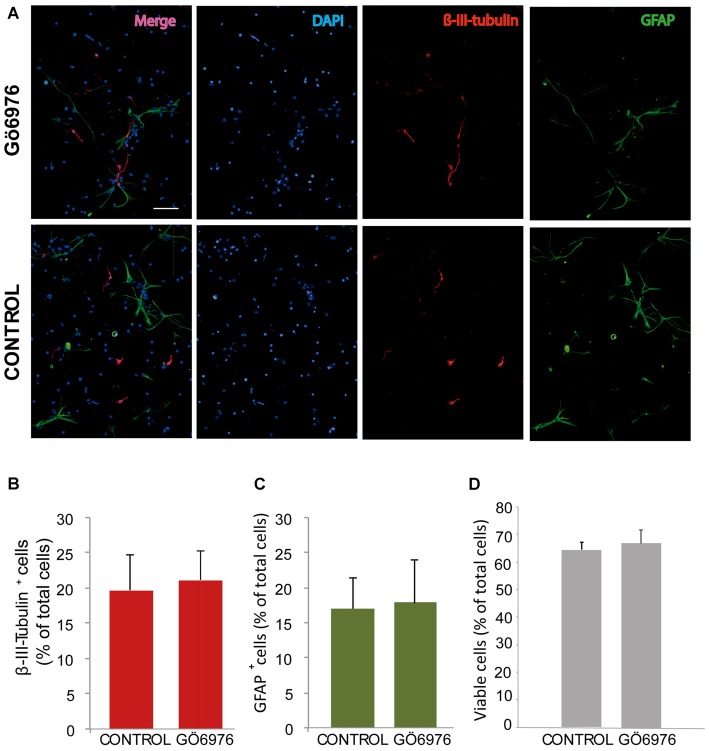
Classical PKC inhibition has no effect on neuroblast or glioblast differentiation. **(A)** Representative fluorescence microphotographs of SVZ-derived cultured neural precursors that had been treated with either diluent (control) or the classical PKC inhibitor Gö6976. Cells were grown in the absence of growth factor and allowed to differentiate for 72 h and then fixed. Neuronal cells were identified by the immunocytochemical detection of β-III-tubulin (red); glial cells are identified by the immunocytochemical detection of GFAP (green) and total nuclei were counterstained with DAPI (blue). Scale bar = 100 μm. **(B)** Graph represents the percentage of total cells (detected by DAPI nuclear staining) that were positive for β-III-tubulin expression. **(C)** Graph represents the percentage of total cells (detected by DAPI nuclear staining) that were positive for GFAP expression. **(D)** Graph represents the percentage of viable cells after treatment as a percentage of total cells (detected by DAPI nuclear staining). Results show no statistically significant changes in the percentage of β-III-tubulin^+^ and GFAP^+^ cells induced by the treatment. Data are the Means ± SEM; *n* = 3 independent experiments performed in triplicates.

**Figure 4:**
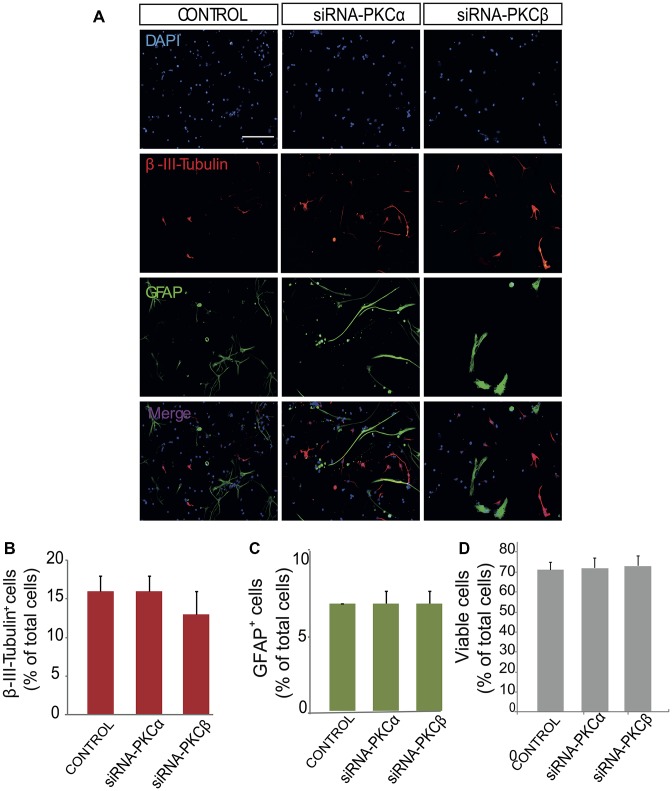
Inhibition of PKCβ and PKCα by siRNA has no effect on neuroblast or glioblast differentiation. **(A)** Representative fluorescence microphotographs of SVZ-derived cultured neural precursors that had been transfected with either mock (control) or the PKCα and PKCβ siRNA. Cells were transfected, grown in the absence of growth factor and allowed to differentiate for 72 h and then fixed with 4% paraformaldehyde (PFA). Neuronal cells were identified by the immunocytochemical detection of β-III-tubulin (red); glial cells are identified by the immunocytochemical detection of GFAP (green) and total nuclei were counterstained with DAPI (blue). Scale bar = 100 μm. **(B)** Graph represents the percentage of total cells (detected by DAPI nuclear staining) that were positive for β-III-tubulin expression. **(C)** Graph represents the percentage of total cells (detected by DAPI nuclear staining) that were positive for GFAP expression. **(D)** Graph represents the percentage of viable cells after treatment as a percentage of total cells (detected by DAPI nuclear staining). Results show no statistically significant changes in the percentage of β-III-tubulin^+^ and GFAP^+^ cells induced by the treatment. Data are the Means ± SEM; *n* = 3 independent experiments performed in triplicates.

**Figure 5:**
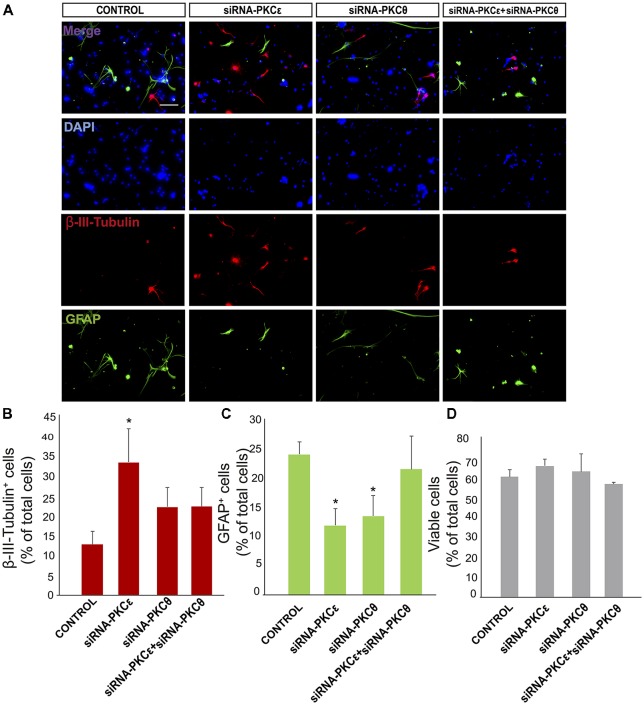
PKCε induces neuronal differentiation *in vitro*. **(A)** Representative fluorescence microphotographs neurosphere-derived adhered cells transfected with PKCε siRNA, PKCθ siRNA, a combination of both siRNA or either mock (control). Neuronal cells were identified by the immunocytochemical detection of β-III-tubulin (red); glial cells are identified by the immunocytochemical detection of GFAP (green) and total nuclei were counterstained with DAPI (blue). Scale bar = 50 μm. **(B)** Graph represents the percentage of total cells (detected by DAPI nuclear staining) that were positive for β-III-tubulin expression after treatments with the different siRNA or mock **(C)** Graph represents the percentage of total cells (detected by DAPI nuclear staining) that were positive for GFAP expression after transfection with the different siRNA or mock (control). **(D)** Graph represents the percentage of viable cells after treatment as a percentage of total cells (detected by DAPI nuclear staining). Results show statistically significant changes in the percentage of β-III-tubulin^+^ and GFAP^+^ cells induced by the inhibition of PKCε and a decreased of GFAP^+^ cells after inhibition of PKCθ. Data are the Means ± SEM; *n* = 3 independent experiments performed in triplicates. Statistical analysis: *p < 0.05 by Student’s *t*-test comparing with the control group.

**Figure 6:**
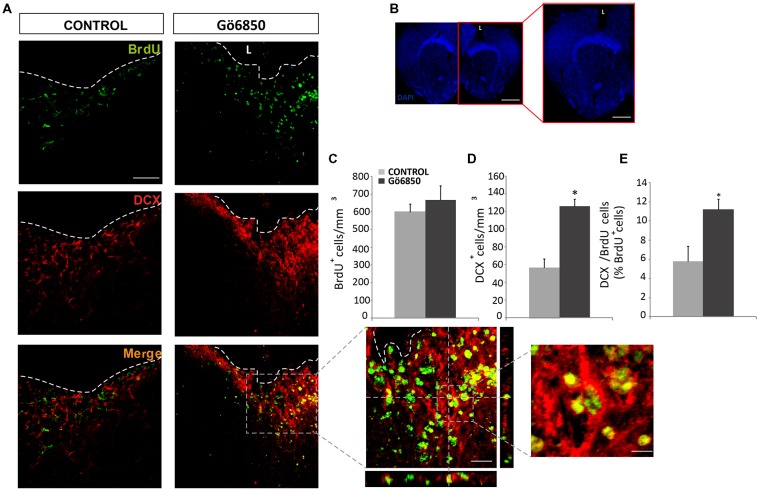
Local inhibition of PKC in the injured cortex increases the number of neuroblast. **(A,B)** Representative confocal microphotographs of the area surrounding cortical lesions in mice locally-infused with vehicle or the general PKC inhibitor Gö6850 (0.5 μM). Mechanical cortical lesions were unilaterally performed in the primary motor cortex of adult mice, and osmotic minipumps were implanted to locally deliver vehicle or Gö6850 for 14 days. All mice were intraperitoneally-injected with bromodeoxyuridine (BrdU) to label proliferating cells. In **(A)** scale bar represent 200 μm in low magnification images, 50 μm in medium magnification images and 20 μm in high magnification images. In **(B)** scale bar represents 2 mm. The dotted line indicates the limit of the lesion (L). **(C)** Graph shows the number of proliferating cells marked with BrdU per mm^3^ in the peri-lesional area of the indicated animal groups. **(D)** Quantification of DCX^+^ cells/mm^3^ in the peri-lesional area of the indicated animal groups. **(E)** Percentage of BrdU^+^ cells that co-expressed DCX in the peri-lesional area. Data shown are the Mean ± SEM; *n* = 3–6 animals per group. Statistical analysis: *p < 0.05 by Student’s *t*-test comparing with the control group.

**Figure 7:**
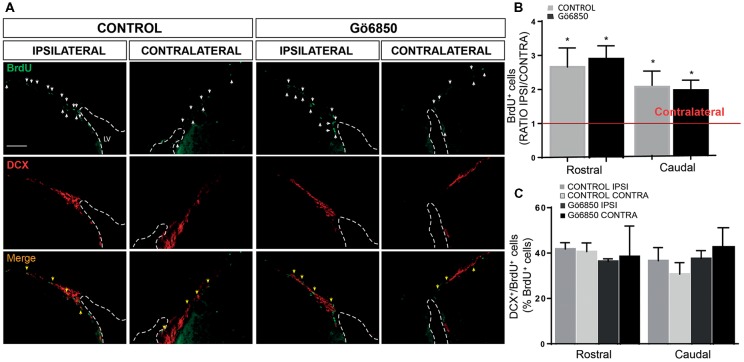
Local inhibition of PKCs has no effect in the neurogenic response of the SVZ to an injury. **(A)** Representative fluorescence microcopy images of the SVZ of adult mice bearing unilateral cortical lesions, locally-infused with vehicle or the general PKC inhibitor Gö6850 (0.5 μM). Cortical lesions, BrdU injections and treatments were performed as described in the legend of Figure 6. Scale bar = 100μm. White arrows indicated BrdU^+^ cells and yellow arrows indicated double-labeled for BrdU and DCX. Dotted lines indicate lateral ventricle walls. **(B)** Graph represents the average ratios obtained when dividing the number of BrdU^+^ cells in ipsilateral SVZs by the number of BrdU^+^ cells in the corresponding contralateral SVZs differentiated in rostral and caudal regions. Statistical analysis: *p < 0.05 when comparing ipsilateral with contralateral SVZs in a Student’s *t*-test for paired samples. **(C)** Percentage of BrdU^+^ cells that co-express DCX in the rostral and caudal SVZ. Data shown are the Mean ± SEM; *n* = 3–6 animals per group. Statistical analysis: ANOVA and Bonferroni post-test.

**Figure 8:**
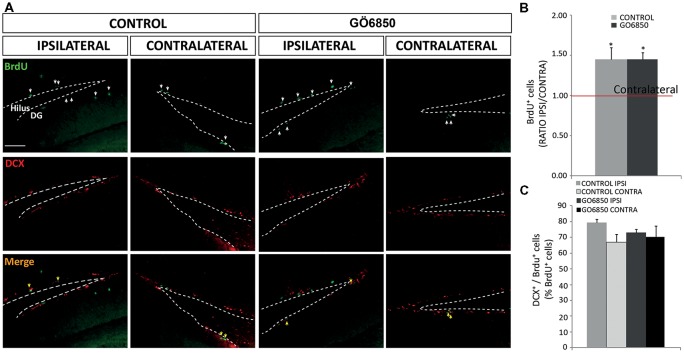
Local inhibition of PKCs has no effects in the neurogenic response of the dentate gyrus (DG) to an injury. **(A)** Representative fluorescence microcopy images of the DG of the hippocampus of adult mice bearing unilateral cortical lesions, locally-infused with vehicle or the general PKC inhibitor Gö6850 (0.5 μM). Cortical lesions, BrdU injections and treatments were performed as described in the legend of Figure 5. Scale bar = 100 μm. White arrows indicated BrdU^+^ cells and yellow arrows indicated double-labeled for BrdU and DCX. Dotted lines indicate the DG border. **(B)** Graph represents the average ratios obtained when dividing the number of BrdU^+^ cells in ipsilateral DG by the number of BrdU^+^ cells in the corresponding contralateral DG. Statistical analysis: *p < 0.05 when comparing ipsilateral with contralateral DG in a Student’s *t*-test for paired samples. **(C)** Percentage of BrdU^+^ cells that co-express DCX in the DG. Data shown are the Mean ± SEM; *n* = 3–6 animals per group. Statistical analysis: ANOVA and Bonferroni post-test.

**Figure 9:**
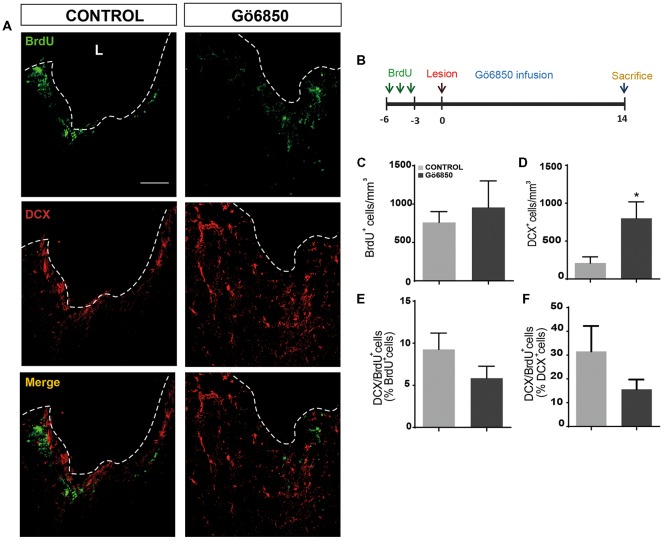
Local inhibition of PKCs has no effect on neural precursors cells migration from the neurogenic regions to the damage area. **(A)** Representative confocal microscopy images of the injured cortex of adult mice after bearing cortical lesions and locally infused with the general inhibitor of PKC Gö6850 (0.5 μM) or only vehicle for 14 days. The cortical injuries were performed to the gray matter in the primary motor cortex. The dotted line indicates the limit of the lesion (L) and the scale bar represent 100 μm. **(B)** Scheme of BrdU administration. Experimental procedure followed to label proliferating neural precursors with BrdU exclusively in neurogenic niches and not in the injured area: mice received BrdU injections on days 6, 5 and 4 before performing the cortical injury; then we waited three more days to allow for complete withdrawal of BrdU from the animal organism. Lesion was performed on day 0 and locally infusion of Gö6850 (0.5 μM) or only vehicle for 14 days. **(C)** Graph shows the number of proliferating cells marked with BrdU per mm^3^ in the peri-lesional area of the indicated animal groups. **(D)** Quantification of DCX^+^ cells/mm^3^ in the peri-lesional area of the indicated animal groups. **(E)** Percentage of BrdU^+^ cells that co-expressed DCX in the peri-lesional area **(F)** Percentage of DCX+ cells that co-localize with BrdU. Data shown are the Mean ± SEM; *n* = 3–6 animals per group. Statistical analysis: *p < 0.05 by Student’s *t*-test comparing with the control group.

### Gö6850 Infusions

In the same surgical act in which the cortex was injured, animals were prepared for a chronic Gö6850 infusion locally within the lesion. Alzet osmotic mini-pumps (Charles River Spain, Barcelona, Spain^3^) were implanted subcutaneously in 12 animals and connected to infusion cannulas (Brain Kit II, Alzet) whose tips were placed 0.5 mm deep into the lesion. This allows a continuous delivery of a 0.5 μM solution of Gö6850 in PBS (containing 0.4% DMSO) or vehicle. Treatment lasted for 14 days post injury (Alzet, 1004). In addition, mice always received three intraperitoneal injections (100 mg/Kg each) of the thymidine analog BrdU on day 14. Each BrdU dose was separated from the next by a 3-h-time interval. The group of animals (vehicle; *n* = 6 and Gö6850-treated mice; *n* = 6) was sacrificed on day 14, 3 h after the last BrdU injection.

### Brain Processing and Immunohistochemistry

Brain removal, processing, sectioning and immunohistochemical detection of BrdU and other cell markers were performed as previously described (Moreno-López et al., 2004; Rabaneda et al., 2008). Primary antibodies used were: mouse monoclonal anti-BrdU (1:100) from Dako (Glostrup, Denmark), goat polyclonal anti-doublecortin (1:200) from Santa Cruz Biotechnology (Santa Cruz, CA, USA) Secondary fluorescent antibodies used (1:1,000) were all from Invitrogen (Carlsbad, CA, USA): donkey anti-goat IgG conjugated to AlexaFluor 594 and donkey anti-mouse IgG conjugated to AlexaFluor 488.

### Stereology and Quantitative Analyses

Stereological methods for unbiased cell counting were used to estimate the number of positive cells expressing the analyzed markers (West, 2002; Hosseini-Sharifabad and Nyengaard, 2007), and 5–6 animals were used per condition.

In the case of the neurogenic regions SVZ and DG, ipsilateral and contralateral sides of the brain (in relation to the cortical lesion) were counted separately; quantifications were done in 1 out of every five 30-mm-thick serial coronal sections spanning, in the rostrocaudal axis in relation to Bregma, from +1.54 mm to −0.94 mm for the SVZ, and from −0.94 mm to −3.64 mm for the DG (Franklin and Paxinos, 1997). In each section, cell number was counted throughout the entire lateral walls of the lateral ventricles (for the SVZ), or the entire DG. The absolute number of cells positive for a given marker in these structures was defined as: *N = ΣQ × 1/ssf*, where ΣQ is the total number of cells counted in all the sections for that structure and *ssf* is the fraction of sections counted (1/5).

To analyze cell number in the lesion perimeter, 3–5 sections containing the cortical injury were selected per mice. In each section, cells positive for the desired markers were quantified within a 200-mm-wide band of tissue adjacent to the lesion border, and the area occupied by this tissue band was also measured using the ImageJ software. Cell density (number of cells counted divided by lesion area and by section thickness) was calculated for each section, and averaged for each animal. Inter-animal differences in the size of perilesional areas were minimal (30 ± 4 × 10^3^ mm^2^/section).

### Statistical Analysis

When more than two treatment groups were compared, statistical analyses were performed using one-factor ANOVA, followed by *post hoc* Bonferroni. The Student’s *t*-test was used when only one treatment group was compared with the control. When ipsilateral vs. contralateral brain hemispheres were compared, the Student’s *t*-test for paired samples was used. The U-Mann Whitney test was used in the case of non-parametric distribution of samples. Differences were considered significant at values of *p* < 0.05.

## Results

### Inhibition of PKC Activity Promotes Neuronal Differentiation of NPC *in vitro*

In order to analyze whether PKC activity had a role in determining the fate of NPCs towards a neuronal lineage, NPCs were cultured *in vitro* and attached onto PLO coated plates in the absence of GFs to facilitate differentiation. The effect of PKC activity on NPC differentiation was tested in these cultures in the presence and absence of the PKC inhibitor Gö6850. After a 72 h treatment period the percentages of neuroblasts (β-III-tubulin^+^ cells), GFAP^+^ cells (astrocytes and NSC), nestin^+^ (undifferentiated progenitors), S100ß^+^ (mature astrocytes) and NG2^+^ (oligodendrocytes) were quantified. In control cultures the percentage of GFAP^+^ cells and β-III-tubulin^+^ cells was similar; around 15%–20% of cells were GFAP^+^ and 15% of them were β-III-tubulin^+^ cells (Figures 1A–C). Inhibition of PKC activity in cultures increased the percentage of β-III-tubulin^+^ cells in 2-fold, reaching almost 35% (Figures 1A,B) whereas the percentage of GFAP^+^ cells remained unchanged (Figures 1A,C). No effect on cell viability was observed (Figure 1D). The percentage of S100ß^+^ astrocytes was around 12% whereas NG2^+^ oligodendrocyte progenitors in control and treated cultures was around 15%. No effect of the treatment on any of these cells was observed (Figures 2A,B). As expected, the percentage of nestin^+^ cells was very small (lower than 4%) in both control and treated cultures. However, nestin^+^ cell number was not altered by the treatment with the PKC inhibitor (Figures 2C–E). These results demonstrated that abolition of PKC activity favored the generation of neurons over astrocytes or oligodendrocytes *in vitro* and suggested that a PKC isozyme that was active in cultures under differentiation conditions was limiting differentiation towards a neuronal fate.

### Expression of PKC Isozymes Under Differentiation Conditions

In order to study the role of PKC isozymes on neuronal differentiation in cultures, we started by analyzing the differential expression of PKC isozymes in attached (differentiating) NPC cultures. As shown in Table 1, under differentiation conditions a high expression of most PKC was observed; particularly classical PKCα and PKCβ, novel PKCθ and PKCε as well as the atypical PKCλ and PKCζ were clearly expressed in these conditions and a small almost undetectable expression of PKCγ, PKCδ and PKCη was observed. The analysis of these results indicated that either the classical PKCα and PKCβ, the novels PKCθ, PKCε or the atypical PKCζ and PKCλ could play a role in preventing neuronal differentiation in cultures.

**Table 1:** Protein kinase C (PKC) expression in neural progenitor cell (NPC) cultures under differentiation conditions.

	PKC	ΔCt	Primer used	Amplicon size	Annealing temperature	Relative abundance
Classical PKC	PKCα	18.01 ± 1.40	FW: TGAATCCTCAGTGGAATGAGT	325	55	+
			RW: GGTTGCTTTCTGTCTTCTGAA			
	PKCβ	15.96 ± 0.87	FW:CCCGAAGGAAGCGAGGGCAATGAAG	227	63	++
			RW: AGTTCATCTGTACCCTTCCGCTCTG			
	PKCγ	21.71 ± 1.73	FW: TGAGAGAGTGCGGATGGGCCCC	104	65	− or −/+
			RW: GCAGGCGTCCTGGGCTGGCACC			
Novel PKC	PKCδ	23.11 ± 2.65	FW: GAGGCCTTGAACCAAGTGACCC	138	57	− or −/+
			RW: CTTGCCATAGGTCCAGTTGTTG			
	PKCε	19.35 ± 1.33	FW: CCCATCTGAAGACGACCGATCC	105	60	+
			RW: CGGTTGTCAAATGACAAGGCC			
	PKCθ	15.65 ± 0.05	FW: CCATGTCACCGTTTCTTCGAATC	132	56	++
			RW: TCTGCCCATTTTCTGATTCC			
	PKCη	24.00 ± 2.53	FW: ATGGCCACGTACCTGAGGCAGC	109	65	− or −/+
			RW: GGACGACGCAGGTGCACACTTGG			
Atypical PKC	PKCλ	17.30 ± 0.95	FW: CGTTGGGAGCTCTGACAATC	240	55	+
			RW: ACCTGCTTTTGCTCCATCATG			
	PKCζ	18.67 ± 2.04	FW: AGGAGAAGAGTACGGGTTCAGC	114	57	+
			RW: GTGTTCATGTCAGGGTTGTCCG			

*Measurement of the mRNA expression of classical, novel and atypical PKC in NPC cultured on differentiation conditions (72 h adhered onto PLO in the absence of growth factors). Total RNA was isolated from differentiated cells derived from neurosphere cultures and subjected to reverse transcription and real-time qPCR. The mRNA for PKC were measured and normalized relative to the levels of 18S rRNA. The table shows ΔCt values (ΔCt = Ct (target mRNA) − Ct (Housekeeping). Abundance is related to this value*.

### Specific Inhibition of Classical PKCα and β Does Not Affect NPC Differentiation

In order to analyze the role of classical PKC on NPC differentiation, a PKC inhibitor Gö6976 with higher specificity for classical PKCs was used in adhered differentiating cultures to inhibit classical PKC during 72 h (Figure 3). No effect on neuronal or glial differentiation was observed in Gö6976 cultures compared to control (Figures 3A–C) and no effect was observed on cell viability (Figures 3A,D). These results indicated that classical PKCs may not be participating in neuronal differentiation *in vitro*. However, PKCα and ß were very abundant in our cultures and therefore it was relevant to elucidate whether inhibition of PKCß and PKCα individually delivered similar results. Therefore, it was analyzed the percentage of neurons and glial cells in cultures transfected with siRNA targeting either PKCα or β during 72 h. Results showed no differences between control cultures (Figures 4A–C) and cultures in which PKCα or PKCβ expression had been abolished. No effect of the siRNAs was observed on cell viability (Figures 4A,D). In addition, two different concentrations of the PKCβ inhibitor hispidin were tested on differentiating cultures for 72 h, the percentage of neurons and glial cells was similar to that found in control cultures (Supplementary Figure S1). This indicated that despite its high expression levels PKCα and ß were not involved in neuronal differentiation under the conditions tested *in vitro*.

### Inhibition of PKCε Expression Facilitates Neuronal Differentiation and Reduces Glial Differentiation *in vitro* in NPC Cultures

We next analyzed whether novel PKC isozymes were responsible for the observed effect on neuronal differentiation. Thus, expression of PKCε was reduced nearly 75% using specific siRNA without affecting expression of other PKC isozymes (Supplementary Figure S2A) or cell viability (Figure 5D). Transfection of cultures with siRNA against PKCε previous to culturing cells under differentiating conditions resulted in a resulted in 2.5-fold increase compared to the control (35% treated vs. 15% control; Figures 5A,B) and surprisingly in a 35% reduction in the percentage of GFAP^+^ astrocytes (Figures 5A,C). This indicated that PKCε played a role in NPC differentiation towards glial lineages, and its inhibition favored neuronal differentiation whereas it reduced glial cells differentiation.

It was next analyzed whether reduction of PKCθ expression exerted an effect on determining NPC fate *in vitro*. Expression was inhibited by transfecting NPCs with siRNA against PKCθ before they were seeded attached onto a substrate for 72 h in the absence of GFs. PKCθ expression was reduced by 50% without affecting other PKC isozymes (Supplementary Figure S2B) or cell viability (Figure 5D). Transfection of cultures with PKCθ siRNA resulted in a reduction in the percentage glial cells similar to that of PKCε silencing (Figures 5A,C), whereas the percentage of neurons remained unchanged (Figures 5A,B). Interestingly, the combination of PKCθ siRNA with PKCε siRNA reverted the effect of the latter suggesting an antagonic effect between the two PKCs isozymes on neuronal differentiation of NPCs (Figures 5A–C).

### Differential Expression of PKC in Cortical Lesions

In order to elucidate whether inhibition of PKCs could be relevant to modulate neurogenesis in brain injuries, we started by analyzing the presence or absence of PKC isozymes in a model of brain injury that we had previously characterized in our laboratory. Lesions consisted in 2 mm deep controlled ipsilateral injuries in the primary motor cortex of mice which affected the gray matter; injuries reached up to the corpus callosum without damaging it (Figure 6B). Expression of PKC isozymes was measured in the injured cortex (ipsilateral) 3 dpi. We observed expression of classical PKCα, PKCβ, PKCγ, novel PKCε, PKCθ and PKCδ and atypical PKCζ expression was detected being PKCθ and PKCδ, the most abundant isozymes (Table 2).

**Table 2:** PKC expression in NPC cultures under differentiation conditions.

PKC	ΔCt	Abundance
PKCα	15.51 ± 4.96	+
PKCβ	8.40 ± 4.52	+
PKCγ	13.47 ± 0.80	+
PKCδ	16.77 ± 0.77	+++
PKCε	10.09 ± 3.92	+
PKCθ	9.91 ± 0.28	++
PKCζ	10.59 ± 4.58	+

*Measurement of the mRNA expression of classical, novel and atypical PKC in injured cortical tissue. Total RNA was isolated from the perilesional tissue, subjected to reverse transcription and real-time qPCR. The mRNA for PKC was measured and normalized to the levels of 18S rRNA. The table shows ΔCt values (ΔCt = Ct (target mRNA) − Ct (Housekeeping). Abundance is related to this value*.

### Local Inhibition of PKC Promotes Neuroblast Enrichment in Brain Lesions

In light of the expression data, it was analyzed whether inhibition of PKC activity in brain injuries led to the generation of neuroblasts and neurons. Thus, we performed mechanical injuries in the motor cortex of mouse brains (Figure 6B). Neurogenesis around the lesion perimeter was analyzed in mice implanted with osmotic minipumps, which constantly released either vehicle or the general PKC inhibitor Gö6850. The treatment did not modify the extent of the injury and no differences were observed at the end of the treatment on the size of the perilesional areas between control and treated groups (29.87 ± 5.38 mm^3^ vs. 26.99 ± 0.87 mm^3^). In response to the lesion BrdU^+^ cells were found in the peri-lesional area of mice treated with either vehicle or Gö6850 14 dpi, and there were no statistically significant differences between these two groups of mice regarding the number of proliferating cells on day 14 (Figures 6A,C). In the control group, some neuroblasts were found around the peri-lesional area of control mice as a response to the lesion. Interestingly a significant 2.5-fold increase in the number of neuroblasts was found in mice treated with the PKC inhibitor (Figures 6A,D). In addition, the percentage of BrdU^+^ cells co-localizing with DCX (BrdU^+^/DCX^+^) increased by 2-fold in mice treated with the general PKC inhibitor (Figures 6A,E). These results indicated that the presence of the inhibitor increased the number of neuroblasts within the peri-lesional area in response to the injury.

### Local Inhibition of PKC in Lesion Does Not Increase the Number of Neuroblast in the SVZ

Lesions were discrete and affected a localized area of the primary motor cortex up to the corpus callosum without damaging this region. However, it was possible that the inhibitor could be reaching the SVZ, and therefore that the effect of the PKC inhibitor on neuroblasts around the injured area could be a consequence of an increased neurogenesis in the SVZ. Thus, we analyzed next BrdU^+^ and BrdU/DCX^+^ cells within the SVZ. As expected, it was found that in response to the lesion the number of BrdU^+^ cells was significantly increased in the ipsilateral SVZ of both control and treated mice, but no effect of the treatment on the SVZ was observed (Figures 7A,B). Identically the percentage of BrdU^+^/DCX^+^ cells increased in the ipsilateral SVZ of both groups but no increase in the number of DCX^+^ cells could be observed in treated mice compared to controls (Figures 7A,C).

### Local Inhibition of PKC in Lesion Has no Effect on the Number of Newly Formed Neuroblasts Within the DG of the Hippocampus

Likewise, it was next analyzed whether local infusion of the PKC inhibitor could modify hippocampal neurogenesis. Thus, the number of proliferative BrdU^+^ cells was quantified in the DG of the hippocampus in control and treated mice. We observed a 1.5-fold increase in the number of BrdU^+^ cells in the ipsilateral DG of control and treated mice compared to the contralateral DG in both groups. However, as expected, no effect of the treatment was observed (Figures 8A,B). Regarding the percentage of BrdU^+^/DCX^+^ cells we found no differences between the ipsilateral and contralateral DG in control and treated mice, and no effect of the treatment was observed either (Figures 8A,C). These results indicated that cell proliferation increased in response to the injury in the ipsilateral DG, but no effect of the treatment in DG neurogenesis was observed.

### Local Inhibition of PKC Does Not Facilitate Migration of Neuroblasts From the SVZ Towards the Injury

In light of the obtained results, it was not clear whether the PKC inhibitor facilitated migration of neuroblasts from the SVZ towards the injury. To determine if this was the case, mice received BrdU injections 6, 5 and 4 days before injury allowing BrdU to be eliminated from the mouse body for another 3 days before performing the injury (Figure 9B). Using this BrdU administration pattern, before performing the injury, only cells within neurogenic niches incorporated BrdU; additionally, the 3-day break between the last BrdU injection and the injury, guaranteed that BrdU-labeled cells were close to the SVZ or, halfway between the SVZ and the olfactory bulb. At the time of injury, osmotic minipumps were implanted to release either vehicle or Gö6850 locally at the lesion area. Animals were sacrificed 14 dpi and brains were processed to detect BrdU^+^ and DCX^+^ cells. As expected, a higher number of neuroblasts was observed within the perilesional area (Figures 9A,D). We observed migration of BrdU^+^ cells towards the injury in both control and Gö6850 treated mice towards (Figures 9A,B). However, no statistically significant changes were observed in the number of BrdU^+^ cells indicating that PKC inhibition was not facilitating migration. In addition, the percentage of neuroblasts that had incorporated BrdU (BrdU^+^/DCX^+^) was not different in treated mice compared to controls (Figures 9E,F) indicating that this inhibitor did not facilitate neuroblast migration in this type of injuries and suggesting the possibility that inhibiting PKC facilitates differentiation as it was observed *in vitro*.

## Discussion

Strategies aimed to replace damaged neurons in brain injuries rely on the capacity of the injured brain to generate new neurons from NSCs. Growing evidences indicate that injuries activate NSCs in an attempt at repairing the damaged tissue (reviewed in Grade and Götz, 2017). However, despite the capacity of the nervous system to produce new neurons from NSCs, replacement of neurons in injuries is limited varying from 0.2%–10%, depending on the affected area and the size and type of injury (Parent et al., 1997; Liu et al., 1998; Jin et al., 2001; Arvidsson et al., 2002; Nakatomi et al., 2002; Teramoto et al., 2003; Romero-Grimaldi et al., 2011; Saha et al., 2013; Moraga et al., 2014). This limited capacity of the CNS to produce new neurons in response to injuries is due in a great extent to the generation of a glial scar within the injured tissue. NPC in injuries have been reported to generate glial cells (Seidenfaden et al., 2006), which contribute to the formation of the glial scar (Sofroniew and Vinters, 2010; Kawano et al., 2012). Cells forming the glial scar secrete signaling molecules that prevent neuronal migration towards the injury (Fawcett and Asher, 1999; Silver and Miller, 2004; Carulli et al., 2005). Additionally, inflammation draws microglial cells towards the injury (Rhodes et al., 2003), which secrete paracrine signaling molecules, regulate function of glial cells, and contribute to a reduction in neuronal survival (Marín-Teva et al., 2011; Benarroch, 2013). This array of cells and paracrine signals generate a gliogenic/non-neurogenic environment around the injured area impairing the generation of new neurons. Therefore, despite the potential of the CNS tissue to produce neurons, they are not produced in injuries unless the gliogenic/non-neurogenic environment is altered by treatments targeting key molecules participating in the regulation of these gliogenic pathways.

In this study, we have hypothesized that a PKC isozyme might be participating in one or more of these gliogenic pathways, and that inhibiting its activity might facilitate the generation of neuroblasts or survival of newly formed neuroblasts inside the injured tissue. Within this context, we have demonstrated that, general PKC inhibition promotes differentiation of NPC towards a neuronal lineage. Based on the obtained results, this effect is partially mediated *in vitro* by the specific inhibition of the novel PKC isozyme, PKCε. In addition, we have determined that several PKC isozymes are expressed in brain injuries and that local treatment of mechanical brain injuries of the primary motor cortex with the general PKC inhibitor Gö6850 promotes the generation of neuroblasts around the peri-lesional area.

First, we demonstrate, using an *in vitro* model of NPC cultures, that general PKC inhibition promotes NPC differentiation towards a neuronal lineage without affecting differentiation towards astrocytes. Treatment of adhered NPC cultures with the general PKC inhibitor, in the absence of GF, increased the percentage of newly formed neurons compared to control cultures, whereas no effect was observed in the percentage of newly formed glial cells. This indicated that a PKC isozyme was preventing neuronal differentiation in cultures and that inhibition of this specific isozyme was facilitating the production of neuroblasts and neurons. The effect was not reproduced in cultures treated with a specific inhibitor of the classical PKC isozymes under identical conditions, indicating that the isozymes responsible for this effect *in vitro* did not belong to the subfamily of classical PKCs. These suggested a role for novel PKC in determining neuronal fate. Atypical PKC could not be considered as targets of the inhibitor since specificity of Gö6850 is mostly for classical and novel PKCs.

In an attempt to elucidate whether different PKC isozymes participated in NPC differentiation, we first analyzed the different PKC isozymes expressed in NPCs under differentiation conditions. We find that the classical PKCα and PKCβ, the novels PKCθ and PKCε and the atypical PKCζ and λ could intervene in the process of neuronal differentiation.

Previous reports show that activation of atypical PKCζ promotes neuronal differentiation from NPCs (Wang et al., 2012) however, the inhibitor used had no or little effect on this enzyme. Therefore, this isozyme could not be the one underlying the neurogenic effect observed. It was then necessary to deeply understand how the different isozymes participated in NPC differentiation. Thus, it was used siRNA to block the differentially expressed isozymes PKCα PKCβ, PKCε and PKCθ in NPC cultures under differentiation conditions. Abolition of PKCε expression facilitated NPC differentiation towards neuroblasts and neurons. In addition, the specific inhibition of PKCε expression reduced glial cell differentiation. This result was in agreement with a previous report showing that overexpression of PKCε results in the production of glial cells from NPCs (Steinhart et al., 2007).

There are growing evidences indicating that NSCs are activated in injuries and that this represents an attempt at repairing the damaged tissue (reviewed in Grade and Götz, 2017). However, neuronal repair is limited in the absence of specific treatments since NPCs within injured brain tissue differentiate to produce glial cells (Romero-Grimaldi et al., 2011). We had demonstrated in here that PKCε activity prevented neuronal differentiation, and that blocking its expression promoted the generation of neuroblasts and neurons in NPC cultures. In addition, both PKCε and PKCθ inhibition led to a reduction in the percentage of newly formed glial cells. However, inhibition of both led to no effect on either neuronal differentiation or glial differentiation, suggesting an interaction between the signaling pathways involving these two kinases. In this work, the effect of PKC inhibition in cortical brain injuries was tested using a model of brain injury previously studied in our laboratory (Romero-Grimaldi et al., 2011) and slightly modified to injure the cortex more deeply up to the corpus callosum, but without damaging it (2 mm). In our previous model, we have observed the presence of proliferating NPCs and glial cells 7 and 14 days post injury, however, no newly formed neuroblasts could be found (Romero-Grimaldi et al., 2011) in untreated injuries. In this new injury model, a significant number of neuroblasts was found in control mice, probably as a consequence of neuroblast migration from the SVZ towards the injury. As expected, it is interesting to note, that neurogenesis was increased in both the SVZ and the DG in control mice in response to the lesion. Thus, a neurogenic response to the injury was found in control mice however, not many neuroblasts were located around the peri-lesional area of control mice suggesting that despite this neurogenic response, not many of them reached the injured tissue. Interestingly, local treatment with the general PKC inhibitor dramatically increased the number of newly formed neuroblasts around the peri-lesional area (Figure 6). A neurogenic response was observed in both the SVZ and DG in response to the injury, however, no increase in the number of proliferating cells and neuroblasts was found either within the SVZ or the DG in response to the treatment (Figures 7, 7, 8). However, it was possible that neuroblasts produced in the SVZ of mice treated with the PKC inhibitor were not present in the SVZ 14 dpi because they had migrated towards the injury. In here, we have tried to elucidate whether migration was facilitated by the inhibitor. We found that neuroblasts and NPC migrated from the SVZ towards the injury in both control and treated mice (Figure 9). Nevertheless, migration rate was not different in control and treated mice. This indicated that PKC inhibitor was probably acting locally around the perilesional area favoring neuroblast survival or neuronal differentiation from NPC rather than facilitating migration. In light of the results presented in here it is not possible to elucidate whether PKC inhibition facilitates neuroblasts differentiation locally at the injured region however, it can be inferred that new neurons emerge around the peri-lesional area in the presence of a PKC inhibitor.

We have not been able to determine the PKC isozymes involved in this effect. Previous reports show that PKCα and PKCδ activation promotes shedding of the EGFR ligands TGFα, amphiregulin and HB-EGF in some cases in a process mediated by ADAM17 (Dang et al., 2011, 2013; Kveiborg et al., 2011). Results from our group had demonstrated that activation of the ADAM17/TGFα/EGFR pathway exerts a gliogenic effect and that these triad is overexpressed in injuries (Dang et al., 2011, 2013). It may be possible that the effect of the PKC inhibitor *in vivo*, is mediated by the inhibition of a PKC, related to the activation of the ADAM17/TGFα/EGFR gliogenic pathway. The results obtained in here are not sufficient to elucidate which isoenzyme/s are mediating the neurogenic effect *in vivo*, however, results show that PKCε inhibition *in vitro* plays an important role in the generation of new neurons. Nonetheless, in addition to the PKCε mediated effect, the inhibition of classical PKCs PKCα and PKCβ *in vivo* around the injury might also participate in enhancing the number of neuroblasts around the injury. PKCβ has previously been involved in promoting NPC proliferation in the SVZ of mice, particularly facilitating proliferation of transit amplifying progenitors (Murillo-Carretero et al., 2017). Therefore, it may be reasonable to suggest that the inhibition of PKCβ *in vivo*, could facilitate differentiation. In addition, activation of PKCα has previously been involved in facilitating the ADAM17/TACE mediated release of the EGFR ligand TGFα (Dang et al., 2011, 2013), which facilitates the generation of glial cells in injuries (Romero-Grimaldi et al., 2011; Geribaldi-Doldán et al., 2018). In relation to the role of classical PKC inhibition *in vitro*, we have not found an effect of classical PKC inhibition on neuronal NPC differentiation under those conditions. However, the *in vitro* culture conditions may be different from those generated within the injury: mainly, no GFs are present in the cultures whereas within the injury precursor of TGFα is overexpressed together with ADAM17/TACE (Dang et al., 2011, 2013). Thus, in addition to the *in vitro-*observed differentiating effect of PKCε it is possible that other PKC isozymes were participating *in vivo*. Furthermore, we were able to observe *in vitro* an antagonic effect of PKCε and PKCθ, which suggests that different PKC isozymes might be exerting different effects on the generation of neurons from NPCs.

Taken together, these findings suggest a role for PKCε in determining the glial fate of NPCs *in vitro* and we have found that local treatment of lesions with a PKC inhibitor facilitates the generation of neuroblasts. These results highlight the role of PKC isozymes as possible targets to facilitate the generation of neurons in injuries.

## Author Contributions

FG-B: cell cultures, mouse injuries, immunocytochemistry and immunohistochemistry, quantification and discussion of results. NG-D: cell cultures, mouse injuries, immunocytochemistry and immunohistochemistry, quantification, data analysis, manuscript writing and figure design and discussion of results. SD-G: immunocytochemistry and immunohistochemistry, quantification, data analysis and discussion of results. MC: mouse injuries, immunohistochemistry, discussion of results and manuscript reviewing. MM-C: cell cultures, immunocytochemistry, RT PCR, data analysis, discussion of results, manuscript writing and reviewing. AD-A: RT PCR, data analysis and discussion of results. MD-S: Cell cultures, immunocytochemistry, data quantification, data analysis, discussion of results and manuscript reviewing. CV: cell cultures, immunocytochemistry, quantification, data analysis, discussion of results and manuscript reviewing. CC: experimental design, manuscript conception, discussion of results, manuscript writing and manuscript reviewing.

## Conflict of Interest Statement

The authors declare that the research was conducted in the absence of any commercial or financial relationships that could be construed as a potential conflict of interest.

## Footnotes

^1^www.thermofisher.com/gibco

^2^www.dharmacon.com

^3^www.criver.com
