# Supplementary figures and images for: Protein Kinase C Inhibition Mediates Neuroblast Enrichment in Mechanical Brain Injuries
Source: Front Cell Neurosci. 2018 Nov 27;12:462. doi: 10.3389/fncel.2018.00462 (PMC6277931; doi:10.3389/fncel.2018.00462)

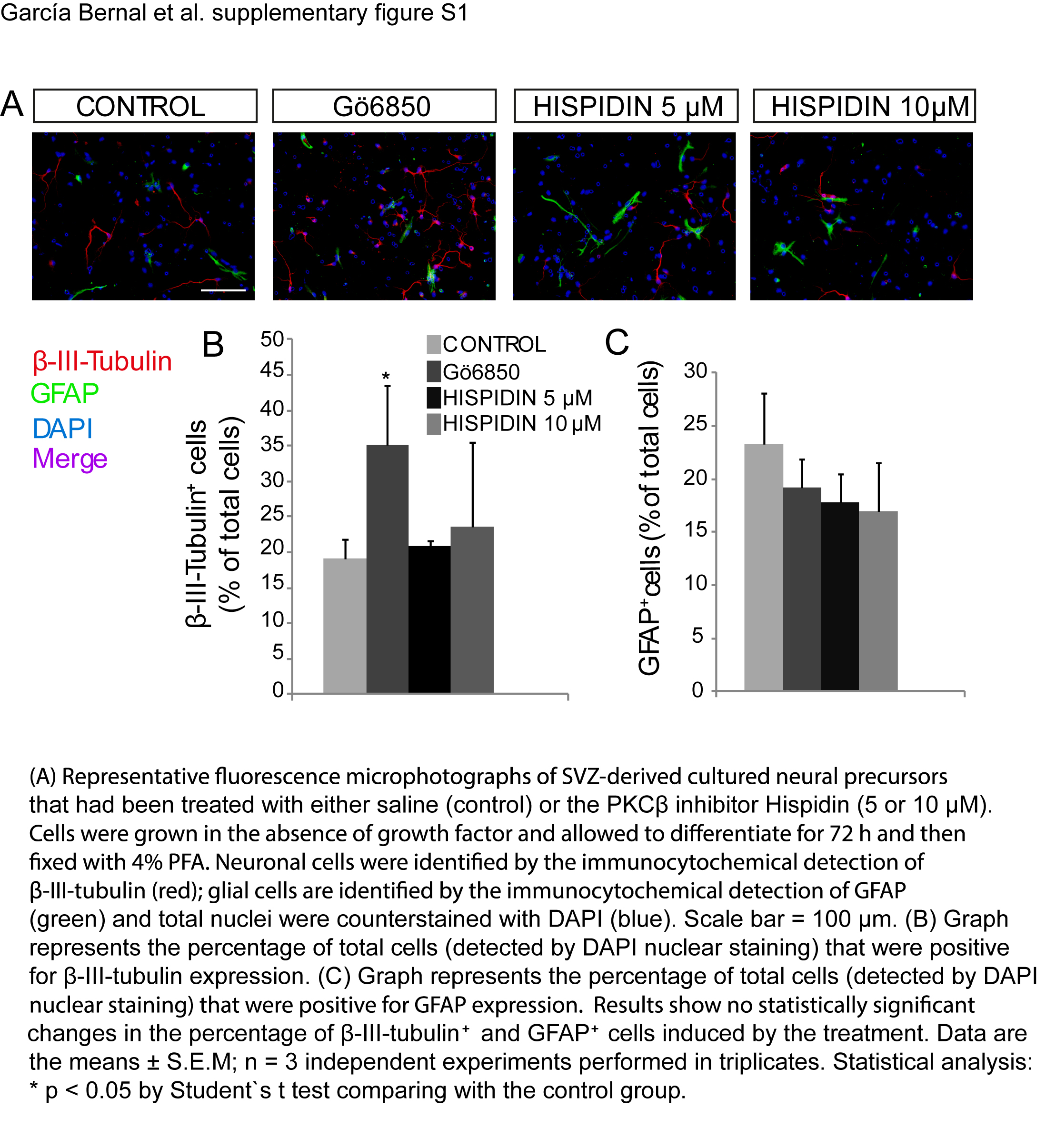

Supplement: Supplementary file 1 [file Image_1.jpg]

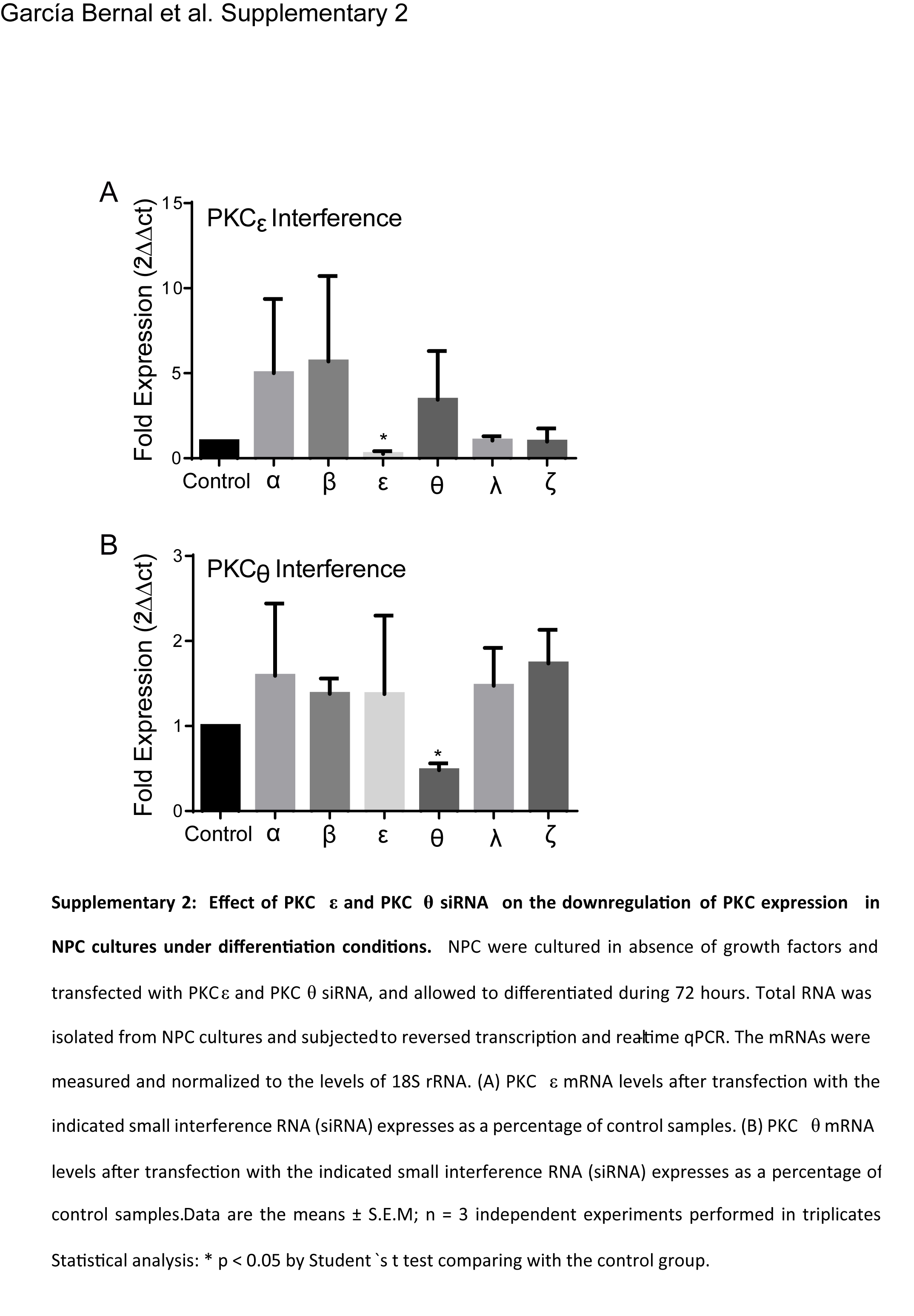

Supplement: Supplementary file 2 [file Image_2.jpg]
